# Peer-assisted debriefing of multisource feedback: an exploratory qualitative study

**DOI:** 10.1186/s12909-018-1137-y

**Published:** 2018-03-14

**Authors:** Jose Francois, Jeffrey Sisler, Stephanie Mowat

**Affiliations:** 1 Department of Family Medicine, Max Rady College of Medicine, Rady Faculty of Health Sciences, University of Manitoba, P219-770 Bannatyne Ave, Winnipeg, MB R3E 0W3 Canada; 2 Continuing Competency and Assessment, Max Rady College of Medicine, Rady Faculty of Health Sciences, University of Manitoba, 260 Brodie, 727 McDermot Ave, Winnipeg, MB R3E 3P5 Canada; 3 Office of Educational and Faculty Development, Max Rady College of Medicine, Rady Faculty of Health Sciences, University of Manitoba, S204-750 Bannatyne Ave, Winnipeg, MB R3E 0W2 Canada

**Keywords:** Multisource feedback, Reflection, Peer-assisted debriefing, Commitment to change, CanMEDS

## Abstract

**Background:**

The Manitoba Physician Achievement Review (MPAR) is a 360-degree feedback assessment that physicians undergo every 7 years to retain licensure. Deliberate reflection on feedback has been demonstrated to encourage practice change. The MPAR Reflection Exercise (RE), a peer-assisted debriefing tool, was developed whereby the physician selects a peer with whom to review and reflect on feedback, committing to change. This qualitative study explores how physicians who had undergone the MPAR used the RE, what areas of change are identified and committed to, and what they perceived as the role of reflection in the MPAR process.

**Methods:**

The MPAR RE was piloted out to a cohort of MPAR-reviewed physicians. Thematic analysis was conducted on completed exercises (*n* = 61). Semi-structured interviews were conducted with individuals (*n* = 6) who completed the MPAR RE until saturation was reached.

**Results:**

Physicians reviewed feedback with a range of peers, including colleagues, staff, and spouses. Many physicians were surprised by feedback, both positive and negative, but interviewees found the RE useful in processing feedback. Areas where physicians committed to change were diverse, covering all CanMEDS roles. Most physicians identified themselves as being successful in implementing change, though time, habit, and structures were cited as barriers.

**Conclusions:**

Peer-assisted debriefing can assist reflection of multisource feedback. It is easy to implement, is not resource-intensive, and feedback implies that it is effective at promoting change. Participants, with the aid of peers, identified areas for change, developed approaches for change, and largely thought themselves successful at implementing changes. Areas of change included all seven CanMEDS roles.

## Background

Physician performance assessments, particularly multisource feedback (MSF) approaches such as 360-degree assessments, are now common requirements for demonstrating continued competence and maintaining certification to practice [1]. In Manitoba, these reviews have been mandatory since 2011; physicians must complete them every 7 years to remain registered to practice in the province. 360-degree assessments are tools whereby physicians are reviewed by colleagues, co-workers, and patients about their performance, as well as completing a self-assessment questionnaire. In Manitoba, the Manitoba Practice Achievement Review (MPAR) is used to collect this feedback, which is then collated into a report that is returned to the physician under review.

The MPAR, based off the Alberta Physician Achievement Review, is a MSF program that provides physicians with a view of their medical practice through the eyes of their medical colleagues, co-workers, and patients, each of whom assess different attributes (Table 1) [2]. The MPAR aims to promote a culture of continuous quality improvement among physicians and is designed to provide feedback in a variety of practice areas using Likert-type questions to gather data about physician performance. MPAR questionnaires cover 15 attributes of practice performance, which are then compared to the average scores of physicians in the province. From this review, those who score within the top 10% in the province are congratulated for their work, while the bottom 10% are examined under further review and may undergo remediation work. However, the remaining 80% are left to their own devices, with no required commitment to reflection, change, or improvement.

**Table 1:** Physician attributes assessed in the Manitoba Physician Achievement Review

Assessor	Attributes assessed
Medical colleague	Medical management Psychosocial management of patients Patient interaction
	Professional self-management Consultation communication
Co-worker	Patient interaction Co-worker collegiality Co-worker communication
Patient	Patient interaction Phone communication Information for patients Personal communication Office staff Physician office Appointments

Assessments such as the MPAR are still relatively new in the province, and while they are well-validated tools, literature on how physicians use this feedback demonstrates that dedicated reflection is required for performance data to change and improve practice, and that physicians will generally not make any changes based on feedback if left to their own devices [3–5]. Indeed, physicians who have been assessed via MSF approaches have identified that having another individual help them reflect on feedback would increase the likelihood of using that feedback [3, 6, 7]. While quantitative data is necessary for rating physicians against average scores, qualitative feedback acquired by reflection, engaging physicians as partners in the MSF process rather than just recipients, is useful for a physician to translate scores into practice change [8] .The studies that have acknowledged the necessity of peer-assisted reflection have generally turned to full peer mentoring or coaching, time-intensive and costly approaches that require trained mentors or coaches who will read a physician’s entire 360-degree report and interview the physician to create a personal development plan [3].

While this approach may be ideal for encouraging practice change, it may not be an appropriate approach for the 80% of physicians in the province who are deemed competent to continue practice after they successfully complete the MPAR; though improvements are encouraged and expected, these successful physicians should not be overburdened with extensive follow-up. To encourage reflection and practice change, the MPAR Reflection Exercise (RE) was developed. This exercise is a peer-assisted debriefing tool, where the physician under review selects a peer, someone familiar with the physician’s practice, with whom to review their MPAR report, after which they fill out the RE, committing to change. Documented reflection and commitment-to-change has been demonstrated to encourage actual practice changes [9, 10].

Peer-assisted learning, where individuals who know each other, and who are not trained educators, are engaged in educational processes, has been demonstrated to allow for more forthcoming communication between the ‘student’ and the peer [11]. Debriefing, a term most often used in crisis management and simulation learning, is appropriate in this context, insofar as it refers to critical reflection with the purpose of resulting in practice change, while having the added benefit of providing an opportunity for building morale and calmly addressing negative or traumatic issues, such as unexpectedly receiving poor feedback [12]. Thus, the collective term ‘peer-assisted debriefing’ implies a productive, formative, casual learning experience. The RE developed by the Continuing Professional Development (CPD) – Medicine Office at the University of Manitoba for peer-assisted debriefing of the MPAR feedback directs and focuses reflection, requiring less time to complete than a full post-assessment interview process. Debriefing can be done with any peer chosen by the physician, and does not require a dedicated, trained mentor to come in and do a full interview.

**Fig. 1:**
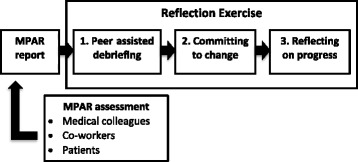
The multisource feedback and reflective process

The MPAR RE is a three-step process following the MPAR assessment (Fig. 1). Briefly, after receiving their MPAR report, the physician selects a peer with whom to review the report. This peer must be someone familiar with the physician and their work, but need not be another physician. The physician must then indicate how they and their peer reviewed the report, and what emerged from this review as important areas of feedback. From there, the physician commits to change in specific changes they will make in response to this feedback and what barriers there may be to making changes. At least 2 months later, the physician reflects on the progress they have made since receiving the MPAR and committing to specific changes. Completion of the RE is eligible for CPD credits. This exploratory study examines completed REs and gathers feedback from individuals who have undergone the MPAR peer-assisted debriefing process with the aim of understanding how the MPAR RE is used, what areas of change are identified and committed, and what they perceived as the role of reflection in the MPAR process.

## Methods

Each year, approximately 200 Manitoba physicians are required to complete the MPAR process. The MPAR RE form is included with the final report that is mailed to physicians following review. Completion of this exercise is eligible for CPD credits from the appropriate crediting body.

The RE has been piloted out to two cohorts of physicians who have completed the MPAR. Completed exercises were examined using thematic analysis to understand how the forms are being used, what areas of feedback are focused on and what changes are committed to [13]. We also examined whom physicians were choosing as their assisting peers and how or whether the changes they identified were carried out.

For more in depth feedback on the debriefing and reflection process, semi-structured telephone interviews were also conducted with individuals who completed the RE. The aim of this interview was to allow participants to share their thoughts on the peer-assisted debriefing process and tool, as well as the MPAR process as a whole.

The coding structure was developed both inductively and deductively; coding of the REs was organized at the top level by the section of the form, and then inductively coded within each section, while interview codes were also organized into deductive topic areas before being coded inductively. SM completed the primary coding of the REs. A research assistant verified the coding structure and completed the primary coding of the interview transcripts, which was then verified by SM. JF then verified the coding of both sets of data in discussion with SM.

This study was approved by the Health Research Ethics Board at the University of Manitoba. Consent was not sought for secondary analysis of completed RE forms; interview participants provided written informed consent.

### Participants

Forms were collected for 2 years, from late 2012 until late 2014, of which 61 (74% male, 26% female; 54% family medicine, 41% other specialties, 5% unspecified) were completed and returned for CPD credits. If approximately 200 Manitoba physicians are reviewed each year, 61 forms over 2 years represents a 15% response rate. Multiple invitations were sent to all 61 of these eligible individuals to participate in the interview process; six interviews were completed. Although the research team had hoped for more interview participants, saturation was reached by the fourth interview. Nonetheless, the generalizability of the interview results must be considered cautiously. All interviewees were male, with 33% in family medicine.

## Results

### Peer-assisted debriefing

**Fig. 2:**
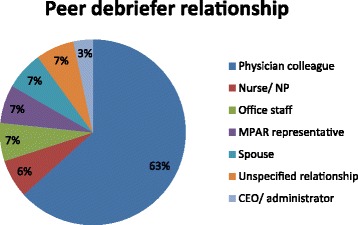
Frequency of peer relationships used in peer-assisted debriefing

This section of the RE required participants to describe their relationship with their peer, describe the process by which they reviewed their MPAR report with their peer, and to discuss their impressions of the MPAR feedback. A variety of relationships were represented among the peer group (Fig. 2). Interviews revealed that many participants chose a peer with whom, whatever the relationship, they felt they could be comfortable sharing personal information:

*“I chose a peer who is somebody that I have known a long time, that I went through medical school with, whose judgement I trust greatly, who I have known from the past just to have good insights into looking at things like this. And somebody that I was comfortable with, and you know, I felt comfortable in sharing this information with him.”*

We had anticipated that most physicians would choose a physician colleague to debrief with; while approximately 60% did choose a fellow physician, 40% chose a different peer. Often this was another co-worker such as a nurse or business manager, which many stated was because they felt this was a person that would be impacted by the changes that needed to be made.

### Committing to change

**Fig. 3:**
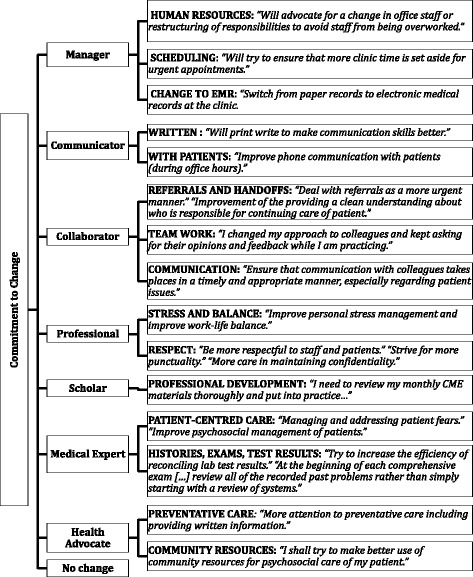
Participant commitment to change statement exemplars, organized by the CanMEDS framework

In collaboration with their peer, respondents developed a plan and committed to change. Themes regarding the commitment to change could be organized quite naturally into the CanMEDS framework, the framework developed by the Royal College of Physicians and Surgeons of Canada that organizes physician areas of competency into seven roles: Medical Expert, Communicator, Collaborator, Professional, Scholar, Health Advocate, and Leader (previously Manager) (Fig. 3) [14]. Areas of change covered much more than just the communicator and collaborator roles that are considered the focus areas of 360 evaluations [15]. Indeed, all of the CanMEDS roles were represented, with many areas of changes identified relating to the organization of practice (scheduling, human resources, electronic records), patient-centeredness, advocacy (use of community resources), professionalism (work-life balance, management of stress, respect), and continuing professional development. Four of the 61 individuals did not believe any changes were necessary based on the feedback they received.

### Reflecting on progress

Two months after committing to change, participants returned to their REs to reflect on the progress they had made. Questions in this section of the tool focused on rating success of change, barriers to change, and any future plans with regard to the areas of change they had committed to. Most participants (73%) felt they were successful in at least some of their chosen areas of change, although many had future plans to improve further. For instance, one physician identified staff communication as an area for improvement; it was a poorly scored area which the physician attributed to poor management of personal stress. In order to address this change, the physician decreased their patient load to reduce stress, and integrated staff meetings into their practice. This process helped the physician identify a further area for change, namely that the practice was understaffed and that there was a need to hire additional staff. Common changes reported included implementation of electronic medical record (EMR) systems to address issues with documentation and scheduling time for urgent same-day appointments and patient calls. One area of change that was often cited as still being in need of improvement was both attending and providing CPD. Time, habit, and structural issues, such as the inability to change physical office space, were commonly cited barriers to changes. Reflecting on the impact that the MPAR process had on their practice, physicians tended to report feeling more confident in the areas in which they had scored well. While there were a small minority of physicians who did not find value in the process, for most, the MPAR merely reinforced that they were overall doing well, with only minor practice changes needed.

### Role of reflection exercise

In interviews, we asked participants to discuss the role of the RE and the peer in facilitating reflection. The peers chosen by the interviewees included physician colleagues, nurses and an MPAR representative. All interviewees had identified area of change including, for instance, improving documentation, improving timeliness of referral responses, providing CPD to colleagues, changing scheduling procedures, and implementing electronic medical records. All interviewees reported at least some success in implementing changes, though some identified areas of further change as they tried to break old habits or make changes with other staff members (e.g., improving staff communication with patients). Interview data were organized into themes of: comparing perspectives; structure for reflection; and approachability and vulnerability.

#### Comparing perspectives

Most seemed to consider that the role of the peer was to supply a different, perhaps more objective, perspective on their MPAR report.

*“But just the opportunity to be able to talk to somebody, and if there was anything, and then sometimes that perspective is like, oh, I hadn't actually thought of it that way, or, you know, pointing out, well as much as you thought that score was a little lower, it actually fits with what everybody else thought you did too, so. So that objective eye.”*

One physician stated how it made him think from the patient’s point of view, making him think of the consequences for patients when he made practice changes.

#### Structure for reflection

All participants admitted that the primary motivation for completing the RE was for the CPD credit. That said, participants who initially did not find a lot of value in the MSF process found that reflecting on the report with a peer helped them process the feedback. The guided reflection gave a structure for reviewing reports.

*“I can flip though the report, but having the reflection tool actually gave me a framework to go back to it and say - so it did help. Because it gave me a little more of a systematic approach to the report, rather than just kind of flipping through the pages and comparing where did the flag go up, where did the flag go down? I can kind of analyze it a little more specifically.”*

#### Approachability and vulnerability

Importantly, participants seemed to appreciate that the debriefing helped them work through their feedback apart from the MPAR process itself, without any pressure or expectations.

*“...you start with the college, it is a little bit intimidating, and it is at least it was to me. You know there is a certain amount of vulnerability where you ask people to let you know what you think about the work you do, and about the way you approach your work, and you are kind of leaving yourself open to criticism or comment. And so it was interesting to get another person's perspective about feedback that I got. I think that the reflection exercise forces you to look at the information that you are provided a little more carefully and closely than you would otherwise. I could see having received the report, thumbed through it, and thought “Gee I think it is mostly pretty good and be happy with that. But the reflection exercise forces you to look at it a little more closely.””*

Even those who did not feel any changes were necessary found the reflection a useful exercise to complete. Reflecting gave physicians a chance to really consider how they felt about the feedback they had received, and most were very reassured by this feedback about how they practiced.

## Discussion

This study represents an important contribution to understanding whether physicians use performance assessment feedback to improve their practice. Dedicated reflection has been demonstrated to be required for performance data to change and improve practice, as physicians will generally not naturally make changes based on feedback alone. Peer-assisted debriefing can facilitate this reflection, and is similar to other peer-based reflection processes observed in other contexts [16]. It is easy to implement, is not resource-intensive, and qualitative feedback implies that it is effective at encouraging change. Debriefing and guided reflection has been shown to be both effective at promoting change and perceived as a valuable exercise among students following simulation activities [17]. Although the context of this study is quite different from studies among students, the evidence indicates that further research in this area is warranted. It is unclear as to whether specific attributes of the physician’s practice type or of the peer selected for the debriefing (such as the difference between a physician and non-physician colleague) impacts on the process or its outcomes.

Participants, with the aid of their peers, identified areas for change, developed an intervention to make changes, and by and large, felt successful at implementing those changes. Reflecting on the process, participants found a more objective perspective helpful, with the exercise providing structure to work through the report of their assessment with a peer without the pressure of the regulatory college, with the incentive of CPD credits. The authors acknowledge that this data is limited insofar as it is a self-selecting sample, using self-reported data with participation incentivized by CPD credits. While this study relies on physician self-reports of change, many did report tangible changes, such as changes to scheduling systems and the implementation of EMRs. Peer-assisted debriefing is likely more than sufficient at encouraging change for the 80% of physicians that are left to process reports completely independently following a multisource feedback review. Moreover, there is some evidence that commitment to change statements can predict actual change in practice [9].

Despite concerns that 360 assessments focus heavily on Communicator, Collaborator, and Professional domains, areas of change identified by participants covered all seven CanMEDS Roles, including Medical Expert, Leader, Scholar, and Health Advocate. It is as yet unclear whether this was based on the report alone, discussions and feedback from the peer, simply the reflection process, or some combination thereof. Further study is warranted into how physicians identified areas for change and, objectively how effective they were in implementing change following peer-assisted debriefing and guided reflection.

## Conclusions

The majority of physicians who completed the peer-assisted debriefing, including reflection and commitment to change, identified areas for change in their practice. These areas covered all the roles a physician plays, as defined by the CanMEDS framework. Interviews with physicians who had undergone the complete process discussed three major themes: comparing perspectives, structure for reflection, and approachability and vulnerability. Though further research is warranted into the nature of the peer relationship and the implementation of changes, the peer-assisted debriefing via a reflection exercise was a means to support physicians in interpreting and making sense of feedback, providing an opportunity for physicians to reflect and process the results of their 360 assessment to plan for continuous improvement.

## Abbreviations

CPD: Continuing professional development
MPAR: Manitoba Physician Achievement Review
MSF: Multisource feedback
RE: Reflection exercise
